# Giant para-anastomotic aneurysms following open abdominal aortic surgery: Open surgery or endovascular management? Report of two cases and literature review

**DOI:** 10.1016/j.ijscr.2025.111444

**Published:** 2025-05-16

**Authors:** Javad Salimi, Amirali Ahrabi

**Affiliations:** a Department of Vascular Surgery, Sina Hospital, Tehran University of Medical Sciences, Tehran, Iran; b Sina Trauma and Surgery Research Center, Tehran University of Medical Sciences, Tehran, Iran

**Keywords:** Abdominal aortic aneurysm, Case report, Endovascular aneurysm repair, FEVAR

## Abstract

**Introduction and importance:**

Para-anastomotic aneurysms (PAAs) represent a late complication of open surgical repair for occlusive or aneurysmal disease of the abdominal aorta. Due to limited literature and management challenges, we present two cases of PAAs and review existing evidence.

**Case presentation:**

Case 1: A 66-year-old man with a history of open infrarenal aortic aneurysm repair presented with mild abdominal pain and a pulsatile mass. Computed tomographic angiography (CTA) showed a juxta-renal PAA near the prior proximal anastomosis. The pseudoaneurysm was managed through fenestrated endovascular aortic aneurysm repair (FEVAR) with four fenestrations.
Case 2: A 76-year-old man with a history of open repair for aortic occlusive disease presented with chronic abdominal pain. CTA revealed two PAAs at the distal anastomosis of the prior aortoiliac graft. The patient underwent open repair surgery.

**Clinical discussion:**

The FEVAR approach avoids redo laparotomy, reducing risks of blood loss, and splanchnic injury. However, prior open repair requires careful endograft design, considering the graft's configuration, reduced compliance, and altered visceral anatomy. For distal anastomotic aneurysms, open repair was chosen due to fewer challenges from adjacent structures.

**Conclusion:**

Based on our experience, FEVAR is effective for juxta-renal PAAs near the proximal anastomosis, while open repair is preferred for PAAs at the distal anastomosis. Further large-scale, long-term studies comparing these treatments are warranted.

## Highlights

• Para-anastomotic aneurysms are a late complication of open surgical repair for abdominal aortic diseases.
• The surgical management of para-anastomotic aneurysms is equivocal and influenced primarily by their location.
• Open repair surgery is one treatment option, though it is technically demanding due to previous surgery.
• EVAR, CHEVAR, & FEVAR offer minimally invasive options when anatomy allows, expanding treatment choices.
• FEVAR is effective for juxta-renal PAAs near the proximal anastomosis; open repair is preferred for distal anastomosis.

## 1 Introduction

Para-anastomotic aneurysms (PAAs), characterized by aortic dilation >5 mm at the anastomotic site, represent a late complication of open surgical repair for occlusive or aneurysmal disease of the abdominal aorta. They manifest as either true aneurysms or pseudoaneurysms and can lead to high morbidity and mortality rates if diagnosed after rupture. These aneurysms tend to be asymptomatic; hence, diagnosis is usually made using computed tomographic angiography (CTA). Even in asymptomatic cases, surgical treatment is indicated, as conservative management has mortality rates of up to 61 % [1].

The surgical management of PAA is equivocal and influenced primarily by its location, with size also playing a significant role in decision-making. Open repair surgery is one treatment option, though it is technically demanding due to the previous surgery. Moreover, patients needing re-intervention after aortic surgery are typically older and have more comorbidities than those initially treated for aortic diseases. Nonetheless, it is still a good fit in cases where vital structures are not densely situated near the surgical field, such as with distal PAAs.

Endovascular abdominal aortic aneurysm repair (EVAR) and related techniques, such as chimney EVAR (CHEVAR) and fenestrated EVAR (FEVAR), offer a minimally invasive alternative when anatomically feasible, thereby broadening treatment options. This is especially useful in challenging cases, such as proximal PAAs, where the aneurysm encroaches on the visceral vessels [2].

Given the limited reported literature on PAAs and the challenges in their surgical management, we aim to present our experience at the Department of Vascular Surgery, Sina Hospital, Tehran, Iran, with two cases: one treated with endovascular option and the other with open repair surgery. Additionally, we provide a comprehensive review of the existing literature on this topic. Written informed consent was obtained from both patients, and this article is in line with PROCESS and SCARE guidelines, as well as the Helsinki declaration [3,4].

## 2 Case report

### 2.1 Case 1

The patient was a 66-year-old man with a history of hypertension, coronary artery disease with stable angina, myocardial infarction, and subsequent coronary artery bypass graft surgery 18 years ago. He also had a history of fusiform infrarenal abdominal aortic aneurysm, initially presented as lower abdominal pain radiating to the back. A computed tomography (CT) scan showed no evidence of rupture. The patient subsequently underwent open repair surgery for an infrarenal aortic aneurysm, using an aortic tube 18-mm Dacron graft via a transperitoneal approach.

Ten years after this surgery, the patient was admitted to our unit with a one-month history of mild abdominal pain and a pulsatile abdominal mass. Upon admission, the patient had an implantable cardioverter-defibrillator (ICD), and a recent report indicating a left ventricular ejection fraction (LVEF) of 20 %. He was hemodynamically stable, with a blood pressure of 125/75 mmHg and a heart rate of 95 beats/min without fever or chills. A moderately tender pulsatile mass was detected during the abdominal examination, but the laboratory tests were unremarkable.

**Fig. 1:**
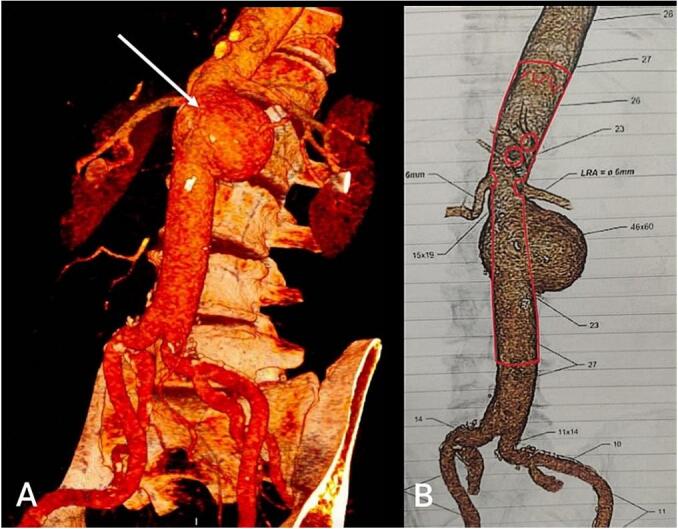
A) CTA showing pseudoaneurysm adjacent to the previous proximal anastomosis of aortoaortic bypass graft (case 1). The juxta-renal position and short landing zone (14 mm to the superior mesenteric artery) make the open repair and other endovascular options such as EVAR and CHEVAR not feasible. B) Planning sketch of FEVAR graft used for case 1.

After ruling out infections, CTA results showed a juxta-renal saccular PAA located next to the previous proximal anastomosis of the aortoaortic bypass graft, measuring 57 mm in maximal diameter with a short landing zone of 14 mm to the superior mesenteric artery (Fig. 1). Due to the presence of adjacent structures in the proximal infrarenal region and the elevated risk associated with open surgery given the patient's cardiovascular comorbidities, open surgery was not considered a viable option. Hence, under general anesthesia, the pseudoaneurysm was managed through fenestrated endovascular aortic aneurysm repair with four fenestrations (4 × FEVAR) using Zenith thoracoabdominal endovascular graft (William Cook Europe ApS, Bjaeverskov, Denmark) extended to the previous abdominal tube graft. The associated visceral arteries and the fenestrations were cannulated, as described by Gallitto et al. [5].

**Fig. 2:**
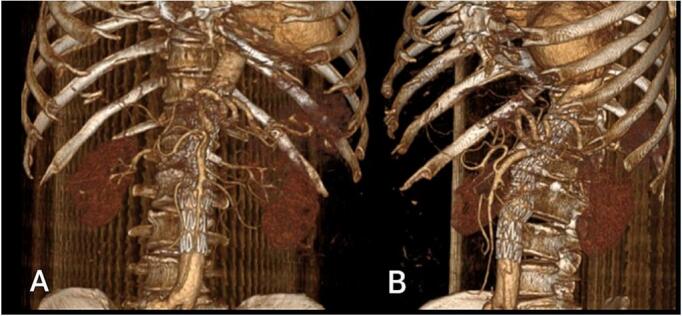
Post-procedural CTA after three months demonstrating the successful exclusion of the pseudoaneurysm with four-fenestrated EVAR performed on case 1. Each target vessel remains patent, with no observed endoleak. A) Anterior view B) lateral view.

**Fig. 3:**
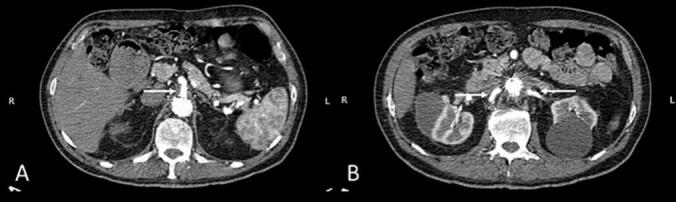
Axial post-procedural CTA image from Case 1 showing a thrombosed juxta-renal pseudoaneurysm adjacent to the fenestrated endograft. A) Patent celiac trunk and superior mesenteric artery are visualized arising anteriorly from the aorta. B) Bilateral renal arteries are patent and arise just proximal to the endograft.

The patient's postoperative recovery was favorable, and the abdominal pain fully resolved. He was discharged on the second postoperative day and has remained under outpatient follow-up for 36 months (Fig. 2, Fig. 3), during which no complaints have been reported.

### 2.2 Case 2

A 76-year-old man, who underwent open surgical repair for abdominal aortic occlusive disease 20 years ago, was admitted to our unit with a two-month history of chronic abdominal pain. He also had a history of chronic hypertension. The patient was hemodynamically stable, presenting with a blood pressure of 135/70 mmHg and a heart rate of 85 beats/min. The patient was afebrile, and laboratory results showed no abnormalities, thereby excluding the possibility of infection.

**Fig. 4:**
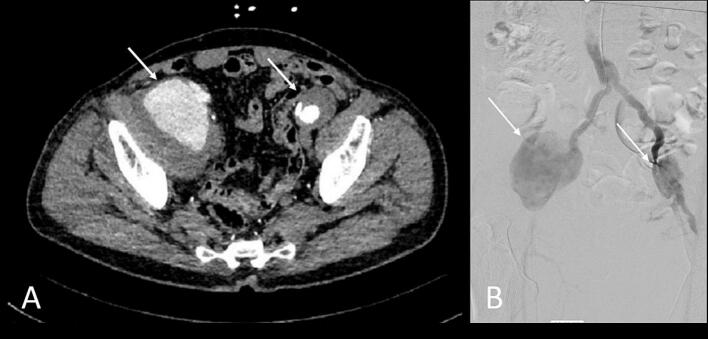
A) Preoperative abdominal CTA showing two distal para-anastomotic aneurysms, indicated by arrows, measuring 70 mm (extending to the right iliac artery) and 45 mm (extending to the left iliac artery) in maximal diameter (case 2), B) the two pseudoaneurysms shown in preoperative digital subtraction angiography.

The main finding on CTA was two pseudoaneurysms measuring 70 mm and 45 mm in maximal diameter in the distal anastomosis of the previous aortic graft to both iliac arteries (Fig. 4). We opted to perform open repair surgery under general anesthesia. Through a midline laparotomy extending above and below the umbilicus, the abdomen was opened. Intra-abdominal adhesions from the previous surgery were mild. The abdominal aorta was then identified.

**Fig. 5:**
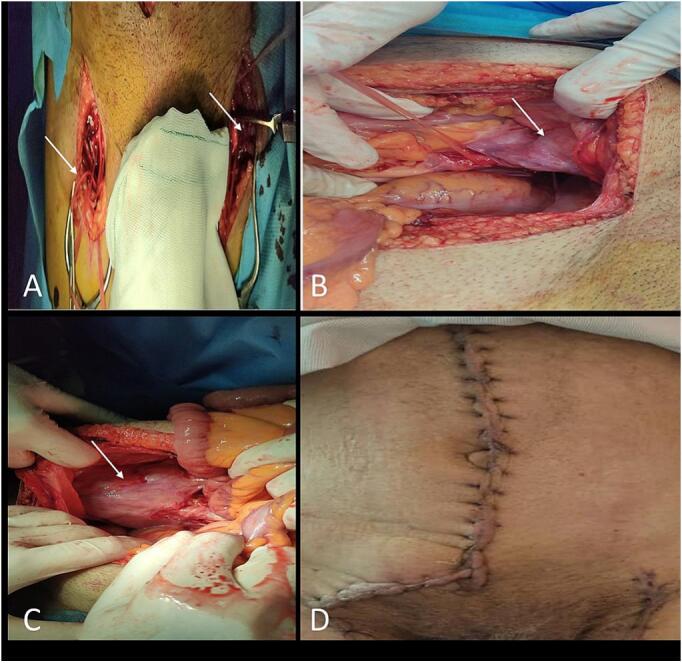
Intraoperative images of open repair surgery in Case 2: A) Bilateral groin incisions for distal control of both femoral arteries; B) proximal control obtained below the bifurcation of the previous graft, with the arrow indicating the left-sided pseudoaneurysm in situ; C) arrow indicating the right-sided pseudoaneurysm; D) final skin closure following completion of the procedure.

Given that the pseudoaneurysms were located at the iliac and femoral anastomosis sites, vascular control was achieved on both the right and left sides of the graft, distal to the graft bifurcation. Additional incisions were made in both femoral regions, allowing bilateral control of the femoral arteries (Fig. 5A).

Subsequently, the pseudoaneurysms were opened on both sides (Fig. 5B and C). A segment of Dacron graft was interposed and anastomosed between the healthy portion of the previous graft and the healthy femoral arteries.

After completing the vascular reconstruction and confirming hemostasis, the abdominal and femoral incisions were irrigated with saline and subsequently closed (Fig. 5D).

The patient was transferred to the recovery room in stable condition. He had an uneventful postoperative recovery and was discharged on the fifth day following surgery. He has remained under outpatient follow-up for 26 months.

## 3 Discussion

One of the complications of abdominal aortic reconstruction is the development of a PAA, which is typically saccular in shape and occurs several months to years post-surgery, with detection at a mean 12 years after operation [6]. These aneurysms are more commonly located at the proximal anastomosis site. Interestingly, Sachdev et al. [7] reported that patients initially treated for aortoiliac occlusive disease took significantly longer to present with PAAs compared to those treated for aneurysmal disease (15.8 vs. 8.9 years, P < .01), which was also observed in our cases.

Surveillance methods and the frequency of CT imaging differ across institutions, and the incidence of PAAs remains poorly defined. In earlier studies, PAAs were typically diagnosed only after fissures, ruptures, or aortodigestive fistulas had occurred. As a result, the prevalence of this condition was believed to be <1 % [8]. However, with increased follow-up, later studies employing active surveillance through imaging modalities have reported an incidence of PAAs reaching up to 27 % over a 15-year period. [6] A recent robust study by Serizawa et al. reported PAA incidence of 2.2 % at five years and 3.6 % at ten years among 147 patients who underwent open repair for abdominal aneurysm of the aorta [9].

Graft infection, especially in the first years after initial repair, can cause secondary aneurysms, so it must be ruled out. The constant degeneration of the arterial wall following graft implantation in addition to the continuous shear stress can weaken the suture between the artery and the prosthesis over time, potentially leading to the formation of a pseudoaneurysm. Hypertension (as observed in both of our cases), along with COPD and tobacco use, are other predisposing factors for the development of PAAs [10,11].

The development of pseudoaneurysm following open repair surgery can also be related to operative strategy and anastomotic technique. Previously, silk suture, end-to-side anastomosis, suture line tension, infection, and multilevel disease have been postulated to be a risk factor for PAA [12]. An end-to-end monofilament suture technique reinforced with Teflon is therefore recommended in open repair surgery [9].

The proposed anatomical criteria by Wu et al. for endovascular treatment of PAA include a proximal aortic neck length >5 mm, a proximal aortic neck angulation of <90 degrees, a proximal aortic neck width of 30 mm, absence of circumferential calcification in the aortic neck or iliac arteries, and an iliac artery diameter of at least 7 mm [13]. Endovascular treatments have an initial technical success rate of 98 % at implantation and reduce perioperative mortality to approximately 3.8 % [14]. Nonetheless, in cases where the proximal aorta is involved, the feasibility of on-label EVAR is <20 % [15]. The advancement of fenestrated, branched, and chimney endovascular techniques has expanded the repertoire of treatments, enabling the management of formidable cases such as juxta-renal aortic pathologies.

The key advantage of the FEVAR approach, which we used in our first case, is that it avoids the need for a redo laparotomy. This minimizes the risk of blood loss, postoperative respiratory failure, and injury to splanchnic organs, resulting in shorter intensive care and hospital stays [2]. However, in cases with prior open repair surgery, additional effort should be made in designing the FEVAR endograft (Fig. 1), taking into account the configuration of the previous graft, its reduced compliance, and the modifications to visceral vessel anatomy. Furthermore, the previous graft may also hinder endograft trackability and rotation when partially deployed.

Standard EVAR or double CHEVAR procedures are not feasible in cases of juxta-renal aneurysms with short landing zones (LZ). As in our case 1, the 14 mm landing zone to the superior mesenteric artery, complicates the ability to anchor the stent graft safely (Fig. 1). On the other hand, t-branch EVAR was not possible due to the small internal lumen diameter (ILD) of the visceral aorta, which would prevent the proper deployment and anchoring of the branched stent graft required to maintain blood flow to the vital visceral arteries such as the celiac, renal, and mesenteric arteries. Accordingly, we opted to perform FEVAR and use the prior surgical graft as the distal sealing zone (Fig. 2). Gallitto et al. [2]. suggested that, in the presence of a safe length between the lowest renal artery and the aortic bifurcation (>4 cm) and with two sealing stents, sealing the FEVAR tube within the surgical graft is preferred in 63 % of their cases. This will ensure that lower limb reperfusion occurs earlier compared to performing a bifurcated implant.

Since the introduction of EVAR, the frequency of open repair surgeries of PAA has been declining, though it continues to be a significant treatment option, particularly in cases that do not meet the anatomical criteria for endovascular treatment or when such procedures are technically challenging. In our second case, the previous graft was completely dissociated from the aortoiliac anastomosis, resulting in large distal pseudoaneurysms (70 mm and 45 mm) that would have prevented the guide wire from advancing from the femoral artery to the proximal part of the graft. Moreover, the distal anastomosis of an aortoiliac graft is not near vital visceral arteries such as the celiac, mesenteric, or renal arteries. Only the presence of adjacent structures like the ureter, inferior vena cava, or iliac veins may give rise to minor challenges. As a result, we opted for open repair surgery in the second case.

Open repair surgery presents significant technical challenges, even in the hands of experienced surgeons, primarily due to adhesions and fibrosis from previous procedures. In our first case, the juxta-renal location of the pseudoaneurysm and involvement of reno-visceral vessels made open repair surgery particularly high risk. Furthermore, the proximal aortic neck, as seen in this case, is typically insufficient for safe infrarenal clamping, often necessitating suprarenal aortic clamping, which increases the risk of renal and visceral complications [16].

Open repair surgery is associated with high morbidity and mortality rates. When performed electively, the mortality rate ranges from 5 % to 17 %, whereas in emergency situations, it increases to 24 % to 88 % [17]. Age over 70 years and a glomerular filtration rate below 48 mL/min per 1.73m^2^ are also significant risk factors for early mortality [18]. Patients who undergo open repair surgery require regular monitoring due to the risk of complications such as renal and respiratory failures.

The precise timing of pseudoaneurysm formation in our cases was uncertain, as the patients did not adhere to the recommended schedule for periodic follow-up. Both the Society for Vascular Surgery and the European Society for Vascular Surgery recommend imaging follow-up every five years after open repair surgery [19,20]. Abdominal and pelvic CT or magnetic resonance angiography are the method of choice. Proper imaging follow-up should never be disregarded, and potentially life-threatening complications in the long term should be anticipated.

## 4 Conclusion

The primary debate in treating these patients centers on whether they should undergo endovascular treatment or open repair. Based on our experience, when a PAA is juxta-renal and adjacent to the previous proximal anastomosis, endovascular treatment—particularly FEVAR—provides an adequate surgical response. In contrast, when the PAA occurs at the distal anastomosis, we prefer to perform open repair surgery due to the less challenging nearby structures. Further large-scale and long-term follow-up studies comparing these two treatment options for PAA after open abdominal aortic repair are warranted.

## CRediT authorship contribution statement

Dr. Javad Salimi designed the concept of the study, collected the data and contributed in data interpretation and writing the paper.
Dr. Amirali Ahrabi contributed in data analysis, interpretation and writing the paper.

## Patient consent

Written informed consent was obtained from both patients.

## Ethical approval

This research was approved by the IRB of our institution.

## Guarantor

Dr. Javad Salimi
Dr. Amirali Ahrabi.

## Patient perspective

Both patients expressed satisfaction with their treatments, noting significant relief from abdominal pain following the procedures.

## Research registration number

This study was not ‘First in Man’.

## Funding

This research received no funding.

## Declaration of competing interest

The authors certify that there is no conflict of interest with any financial organization regarding the material discussed in the manuscript.
